# Healthcare-associated infections in patients with severe COVID-19 supported with extracorporeal membrane oxygenation: a nationwide cohort study

**DOI:** 10.1186/s13054-024-04832-3

**Published:** 2024-02-20

**Authors:** Nicolas Nesseler, Alexandre Mansour, Matthieu Schmidt, Marylou Para, Alizée Porto, Pierre-Emmanuel Falcoz, Nicolas Mongardon, Claire Fougerou, James T. Ross, Antoine Beurton, Lucie Gaide-Chevronnay, Pierre-Grégoire Guinot, Guillaume Lebreton, Erwan Flecher, André Vincentelli, Nicolas Massart, ECMOSARS Investigators, Olivier Fouquet, Marc Pierrot, Sidney Chocron, Guillaume Flicoteaux, Philippe Mauriat, Alexandre Ouattara, Hadrien Roze, Olivier Huet, Marc-Olivier Fischer, Raphel Bellaïche, Ophélie Constant, Quentin De Roux, L. Y. André, Arnaud Meffert, Jean-Claude Merle, Lucile Picard, Elena Skripkina, Thierry Folliguet, Antonio Fiore, Nicolas D’ostrevy, Marie-Catherine Morgan, Maxime Nguyen, Nicolas Terzi, Gwenhaël Colin, Olivier Fabre, Arash Astaneh, Justin Issard, Elie Fadel, Dominique Fabre, Julien Guihaire, Iolande Ion, Jean Baptiste Menager, Delphine Mitilian, Olaf Mercier, François Stephan, Jacques Thes, Jerôme Jouan, Thibault Duburcq, Valentin Loobuyck, Mouhammed Moussa, Sabrina Manganiello, Agnes Mugnier, Natacha Rousse, Olivier Desebbe, Jean-Luc Fellahi, Roland Henaine, Matteo Pozzi, Zakaria Riad, Christophe Guervilly, Sami Hraiech, Laurent Papazian, Matthias Castanier, Charles Chanavaz, Cyril Cadoz, Sebastien Gette, Guillaume Louis, Erick Portocarrero, Philippe Gaudard, Kais Brini, Nicolas Bischoff, Antoine Kimmoun, Bruno Levy, Pierre Perez, Alexandre Bourdiol, Yannick Hourmant, Pierre-Joachim Mahé, Bertrand Rozec, Mickaël Vourc’h, Stéphane Aubert, Florian Bazalgette, Claire Roger, Pierre Jaquet, Brice Lortat-Jacob, Pierre Mordant, Patrick Nataf, Juliette Patrier, Sophie Provenchere, Morgan Roué, Romain Sonneville, Alexy Tran-Dinh, Paul-Henri Wicky, Charles Al Zreibi, Bernard Cholley, Yannis Guyonvarch, Sophie Hamada, Claudio Barbanti, Astrid Bertier, Anatole Harrois, Jordi Matiello, Thomas Kerforne, Corentin Lacroix, Nicolas Brechot, Alain Combes, Juliette Chommeloux, Cosimo D’alessandro, Pierre Demondion, Alexandre Demoule, Martin Dres, Guillaume Fadel, Muriel Fartoukh, Guillaume Hekimian, Charles Juvin, Pascal Leprince, David Levy, Charles Edouard Luyt, Thibaut Schoell, Pierre Fillâtre, Nicolas Massart, Maud Jonas, Nicolas Allou, Salvatore Muccio, Dario Di Perna, Vito-Giovanni Ruggieri, Bruno Mourvillier, Amedeo Anselmi, Karl Bounader, Yoann Launey, Thomas Lebouvier, Alessandro Parasido, Florian Reizine, Maxime Esvan, Philippe Seguin, Emmanuel Besnier, Dorothée Carpentier, Thomas Clavier, Anne Olland, Marion Villard, Fanny Bounes, François Labaste, Vincent Minville, Antoine Guillon, Yannick Fedun, SFAR Research Network

**Affiliations:** 1 Department of Anesthesia and Critical Care, Pontchaillou, University Hospital of Rennes, Rennes, France; 2 Univ Rennes, CHU Rennes, Inserm, CIC 1414 (Centre d’Investigation Clinique de Rennes), 35000 Rennes, France; 3 Univ Rennes, CHU de Rennes, Inra, Inserm, Institut NUMECAN – UMR_A 1341, UMR_S 1241, 35000 Rennes, France; 4 Univ Rennes, CHU Rennes, Inserm, IRSET, UMR_S 1085, CIC 1414 (Centre d’Investigation Clinique de Rennes), 35000 Rennes, France; 5 Sorbonne Université, INSERM, UMRS_1166-ICAN, Institute of Cardiometabolism and Nutrition, 75013 PARIS, France; 6 Service de Médecine Intensive-Réanimation, Institut de Cardiologie, APHP Sorbonne Université Hôpital Pitié–Salpêtrière, 75013 Paris, France; 7 Department of Cardiovascular Surgery and Transplantation, Bichat Hospital, AP-HP, Paris, France; 8 Laboratory of Vascular Translational Science, University of Paris, UMR 1148, Paris, France; 9 Department of Cardiac Surgery, Timone Hospital, APHM, 13005 Marseille, France; 10 INSERM, UMR 1260, Regenerative Nanomedicine (RNM), FMTS, 67000 Strasbourg, France; 11 Faculté de Médecine et Pharmacie, Université de Strasbourg, 67000 Strasbourg, France; 12 Hôpitaux Universitaire de Strasbourg, Service de Chirurgie Thoracique - Nouvel Hôpital Civil, Strasbourg, France; 13 Service d’anesthésie-Réanimation, DMU CARE, DHU A-TVB, Assistance Publique-Hôpitaux de Paris (AP-HP), Hôpitaux Universitaires Henri Mondor, 94010 Créteil, France; 14 Faculté de Santé, Univ Paris Est Créteil, 94010 Créteil, France; 15 U955-IMRB, Equipe 03 « Pharmacologie et Technologies pour les Maladies Cardiovasculaires (PROTECT), Inserm, Univ Paris Est Créteil (UPEC), Ecole Nationale Vétérinaire d’Alfort (EnVA), 94700 Maisons-Alfort, France; 16 Department of Clinical Pharmacology, University Hospital, Rennes 1 University, 35033 Rennes, France; 17 Inserm CIC 1414, Clinical Investigation Centre, University Hospital, Rennes 1 University, 35033 Rennes, France; 18 Department of Surgery, University Hospitals Cleveland and Case Western Reserve University, Cleveland, USA; 19 Department of Anaesthesia and Critical Care, CHU Bordeaux, Magellan Medico-Surgical Centre, 33000 Bordeaux, France; 20 UMR 1034, Biology of Cardiovascular Diseases, Univ. Bordeaux, INSERM, 33600 Pessac, France; 21 Department of Anesthesiology and Critical Care Medicine, University Hospital of Grenoble, Grenoble, France; 22 Department of Anesthesiology and Critical Care Medicine, Dijon University Hospital, Dijon, France; 23 Sorbonne Université, INSERM, UMRS_1166-ICAN, Institute of Cardiometabolism and Nutrition, Paris, France; 24 Service de Chirurgie Thoracique et Cardiovasculaire, Institut de Cardiologie, APHP, Sorbonne Université, Hôpital Pitié–Salpêtrière, Paris, France; 25 Department of Thoracic and Cardiovascular Surgery, Signal and Image Treatment Laboratory (LTSI), Pontchaillou University Hospital, University of Rennes 1, Inserm U1099, Rennes, France; 26 Cardiac Surgery, Univ. Lille, CHU Lille, 59000 Lille, France; 27 Univ. Lille, Inserm, CHU Lille, Institut Pasteur de Lille, U1011-EGID, 59000 Lille, France; 28 Intensive Care Unit, Centre Hospitalier Yves Le Foll, Saint-Brieuc, France; 29 Hôpital Pontchaillou, Pôle Anesthésie, SAMU, Urgences, Réanimations, Médecine Interne Et Gériatrie (ASUR-MIG), 2 Rue Henri Le Guilloux, 35033 Rennes Cedex 9, France

**Keywords:** ECLS, SARS-CoV 2, Nosocomial infections, Ventilator-associated pneumonia, Bloodstream infections

## Abstract

**Background:**

Both critically ill patients with coronavirus disease 2019 (COVID-19) and patients receiving extracorporeal membrane oxygenation (ECMO) support exhibit a high incidence of healthcare-associated infections (HAI). However, data on incidence, microbiology, resistance patterns, and the impact of HAI on outcomes in patients receiving ECMO for severe COVID-19 remain limited. We aimed to report HAI incidence and microbiology in patients receiving ECMO for severe COVID-19 and to evaluate the impact of ECMO-associated infections (ECMO-AI) on in-hospital mortality.

**Methods:**

For this study, we analyzed data from 701 patients included in the ECMOSARS registry which included COVID-19 patients supported by ECMO in France.

**Results:**

Among 602 analyzed patients for whom HAI and hospital mortality data were available, 214 (36%) had ECMO-AI, resulting in an incidence rate of 27 ECMO-AI per 1000 ECMO days at risk. Of these, 154 patients had bloodstream infection (BSI) and 117 patients had ventilator-associated pneumonia (VAP). The responsible microorganisms were Enterobacteriaceae (34% for BSI and 48% for VAP), Enterococcus species (25% and 6%, respectively) and non-fermenting Gram-negative bacilli (13% and 20%, respectively). Fungal infections were also observed (10% for BSI and 3% for VAP), as were multidrug-resistant organisms (21% and 15%, respectively). Using a Cox multistate model, ECMO-AI were not found associated with hospital death (HR = 1.00 95% CI [0.79–1.26], *p* = 0.986).

**Conclusions:**

In a nationwide cohort of COVID-19 patients receiving ECMO support, we observed a high incidence of ECMO-AI. ECMO-AI were not found associated with hospital death.

*Trial registration number* NCT04397588 (May 21, 2020).

**Supplementary Information:**

The online version contains supplementary material available at 10.1186/s13054-024-04832-3.

## Background

Healthcare-associated infections (HAI) are frequent in patients receiving extracorporeal membrane oxygenation (ECMO) support [1, 2]. Likewise, critically ill patients with coronavirus disease 2019 (COVID-19) have a higher incidence of HAI compared to non-COVID-19 critically ill patients or those admitted to intensive care unit (ICU) before the pandemic[3–5]. Both ECMO support and severe acute respiratory syndrome coronavirus 2 (SARS-CoV-2) induce immune alterations that may increase the susceptibility to HAI [6, 7]. A recent European multicenter study reported high incidences of ventilator-associated pneumonia (VAP) and bloodstream infections (BSI) in COVID-19 patients on ECMO [8]. Yet, data on microbiology, resistance patterns, and its impact on the outcomes in patients receiving ECMO for severe COVID-19 remain limited [9]. The primary objective of this prospective multicenter cohort study was to report the incidence and microbiology of HAI in patients receiving ECMO for severe COVID-19. The secondary objective was to evaluate the impact of ECMO associated infections (ECMO-AI) on patient outcomes. We hypothesized that the incidence of ECMO-AI would be high and associated with worse outcomes in patients receiving ECMO for severe COVID-19.

## Methods

### Data collection

The French national Extracorporeal Membrane Oxygenation for Respiratory Failure and/or Heart failure related to Severe Acute Respiratory Syndrome-Coronavirus 2 (ECMOSARS) registry recruited all COVID-19 patients supported by ECMO (Veno-Venous (VV) or Veno-Arterial (VA)) between April 2020 and March 2022 (ClinicalTrials.gov Identifier: NCT04397588) [10]. The registry has been approved by the university hospital of Rennes ethics committee (n° 20.43). According to the French legislation, written consent was waived because of the observational design of the study that does not imply any modification of existing diagnostic or therapeutic strategies. After information, only non-opposition of patients or their legal representative was obtained for use of the data. The data collection methodology has been previously reported [10–12]. Briefly, data were collected by research assistants from each patient’s medical record using an electronic case report form. Automatic checks were generated for missing or incoherent data, and additional consistency tests were performed by data managers. Collected data included patient characteristics and comorbidities, management of COVID-related acute respiratory distress syndrome before ECMO cannulation, patient characteristics at ECMO cannulation and the day after, therapeutics, complications and patient outcomes on ECMO. Patient and ECMO management was at the discretion of each center (see Additional file 1: Table S1 for the definition of the main variables). The strategies for HAI prevention were left to the discretion of each ICU. Center experience was classified in two groups according to their experience in ECMO management before the pandemic: centers that managed more than 30 ECMO patients (≥ 30) annually were considered high volume, and those that managed fewer than 30 ECMO patients (< 30) annually were considered low volume [13].

### Outcomes

Our primary outcome was HAI incidence while on ECMO (ECMO-AI). Secondary outcomes were incidences of VAP and BSI, ECMO-AI microbiology and antimicrobial resistance, ECMO-free days within 90 days of cannulation, ventilatory-free days within 90 days of cannulation, and in-hospital death.

### Definitions

ECMO-AI included both VAP and BSI. An infection was classified as ECMO-AI if it developed during the ECMO run, was diagnosed 48 h or more after ICU admission and was not incubating upon admission. Diagnosis was made by treating physician. Within each subtype of ECMO-AI (VAP or BSI), only the first event was recorded. BSI was defined by a positive blood culture occurring 48 h or more after admission. For common skin contaminants, confirmation required two positive blood cultures drawn from separate puncture site [14]. The diagnosis of VAP was considered in patients ventilated for 48 h or more, and up to 48 h after extubation. The criteria for the diagnosis of VAP followed the current French guidelines [15]. Microorganisms identified as the cause of infection were categorized as multidrug-resistant organisms (MDRO) based on the European Society of Clinical Microbiology and Infectious Disease definition [16]. The first epidemic wave (up to July 1st, 2020) was distinguished from the subsequent waves (from July 1st, 2020, to March 31, 2022).

### Study design and population

For the present study, we analyzed all consecutive patients included in the registry with available data on acquired infections and hospital mortality. The analysis followed the Strengthening the Reporting of Observational Studies in Epidemiology (STROBE) guidelines.

### Statistical analysis

A statistical analysis plan was made prior to accessing the data. No a priori statistical power calculation was conducted. Categorical variables were expressed as number (percentage) and continuous variables as median and interquartile range. When appropriate, the chi-square test and the Fisher’s exact test were used to compare categorical variables. The Mann–Whitney U test and the Wilcoxon test were used to compare continuous variables. Multiple imputations were used to replace missing data. Missing data were assumed to be missing at random and were dealt using “MICE” R package using Monte Carlo Markov chained equations to generate a dataset without missing values. The variable selected to predict missing values was those available before exposure to the risk of HAI (outcome variables not included). To evaluate the association between ECMO-AI and in-hospital mortality, we performed survival analyses using a multivariable proportional Cox model. Given that ECMO-AI developed during follow-up and was not present at cannulation, a multistate model was constructed [17]. As a result, patients who developed an ECMO-AI were included twice. First, they were included in the group without ECMO-AI from cannulation to the onset of ECMO-AI. Then, they were censored from this group and included in the ECMO-AI group from the onset of ECMO-AI to discharge or death. Confounders entered in the multivariable model were defined a priori based on the existing ECMO and COVID-19 literature. All confounders that are associated with both ECMO-AI and death were included in the multivariable analysis. The set of potential confounders sufficient for adjustment was: center case volume, epidemic wave (first vs subsequent), age, diabetes, chronic respiratory failure, chronic kidney disease, malignancy (solid cancer or hemopathy), use of steroids before ECMO, use of non-steroidal anti-inflammatory drugs before ECMO, septic shock, antibiotic before cannulation, selective digestive decontamination, SOFA score at cannulation, type of ECMO support (VA vs VV), delay from hospitalization to ECMO cannulation. All tests were two-sided, and p < 0.05 was considered statistically significant.

## Results

### Study population

**Fig. 1:**
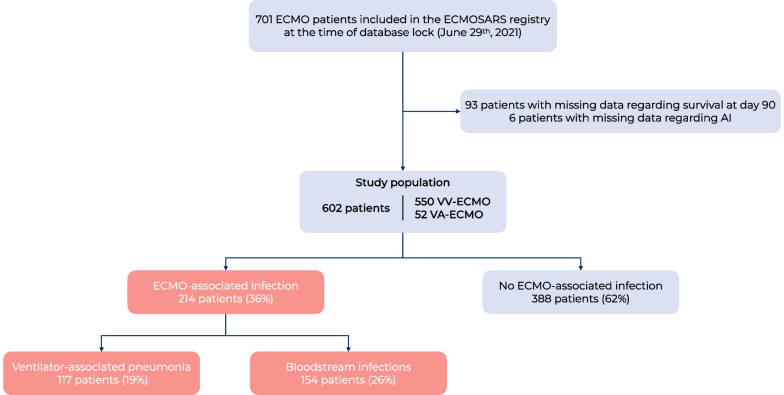
Flow chart of ECMO patients included in the study

Among the 47 participating ICUs, 701 patients were included in 41 ICUs in the registry at the time of database lock. Of these, 6 patients had missing data concerning ECMO-AI and an additional 93 had missing survival data, leaving a total of 602 patients available for analysis (Fig. 1). Most patients (73%) were admitted during first epidemic wave (Table 1). The median age was 55 (46–61) years. Patients were intubated for a median of 5 (2–8) days before cannulation, 541/599 patients (90%) underwent prone positioning and 565/595 patients (95%) received neuromuscular blocking agents before cannulation. The median PaO2/FiO2 ratio before cannulation was 63 (54–77) mmHg. Additionally, 432/477 (91%) received antibiotics before ECMO initiation and 15 (2%) received selective digestive decontamination before ECMO initiation in 4 ICUs. Most ECMO were veno-venous (550/602, 91%). Superior–inferior vena cava was the most common site of cannulation (515/602, 86%), mostly through femoro-jugular access (493/602, 82%).

**Table 1:** Patient characteristics at the time of ECMO cannulation

Characteristics	Missing data Infected/not infected	ECMO-associated infection (n = 214)	No ECMO-associated infection (n = 388)	P Value
Epidemic waves	0/0			0.028
First epidemic wave		149 (69.6)	303 (72.1)	
Subsequent epidemic waves (vs first)		65 (30.4)	85 (21.9)	
Age—years	0/0	56 [48–62]	54 [45–61]	0.064
Male sex	0/0	160 (74.8)	307 (79.1)	0.261
Body mass index – kg/m^2^	4/18	30.85 [27.02–34.77]	29.65 [26.50–34]	0.181
Comorbidities				
Chronic hypertension	0/0	90 (42.1)	144 (37.1)	0.270
Diabetes	0/4	61 (28.5)	118 (30.7)	0.634
Chronic respiratory failure	0/0	7 (3.3)	12 (3.1)	1.000
Chronic cardiac failure	0/48	3 (1.4)	9 (3.4)	0.241
Chronic kidney disease	0/121	9 (4.2)	12 (4.5)	1.000
Onco-hematological malignancy	0/124	5 (2.3)	7 (2.7)	1.000
Clinical, condition and management before cannulation				
Center case-volume	0/0			< 0.001
High		141 (65.9)	320 (82.5)	
Intermediate		56 (26.2)	51 (13.1)	
Low		17 (7.9)	17 (4.4)	
Referral center	0/6	117 (54.7)	218 (56.2)	0.786
Mobile ECMO team, transfer to referral center	1/15	49 (22.9)	130 (34.0)	0.006
Simplified acute physiology score II	0/0	39 [29–53]	32 [24–49]	< 0.001
Lowest PaO2/FiO2 – mmHg	10/25	61.50 [53–78.25]	64 [54–77]	0.781
Treatment before cannulation				
Steroids	0/123	16 (7.5)	18 (6.8)	0.912
Neuromuscular blocking agent	1/4	204 (95.8)	361 (94.0)	0.467
Prone positioning	1/2	197 (92.5)	344 (89.1)	0.234
Noninvasive ventilation	2/3	72 (34.0)	113 (29.4)	0.283
High-flow oxygen therapy	3/121	108 (51.2)	132 (49.4)	0.774
Antibiotic	0/125	194 (90.7)	238 (90.5)	1.000
Penicillin		41 (19.2)	71 (27.0)	0.057
Cephalosporin		158 (73.8)	178 (67.7)	0.173
Macrolides		106 (49.5)	125 (47.5)	0.731
Therapeutic anticoagulation	4/129	102 (48.6)	104 (40.2)	0.083
Selective digestive decontamination	0/0	6 (2.8)	9 (2.3)	0.927
Characteristics at ECMO cannulation				
Delay from intubation to cannulation – days	4/8	5 [2–8]	5 [3–8]	0.577
Veno-arterial ECMO (vs Veno-Venous)	0/0	12 (5.6)	40 (10.3)	0.050
Cannulation site	0/0			0.946
Both superior and inferior vena cava		184 (86.0)	330 (85.1)	
Only inferior vena cava		24 (11.2)	45 (11.6)	
Only superior vena cava		3 (1.4)	5 (1.3)	
Others/unknown		3 (1.4)	8 (2.1)	
PaO2/FiO2 – mmHg	10/19	68 [59–85]	67 [55–85]	0.325
SOFA score	0/0	8 [5–11]	10 [8–12]	< 0.001
Norepinephrine	4/130	117 (55.7)	165 (64.0)	0.086
Renal Replacement Therapy	0/8	23 (10.7)	48 (12.6)	0.584
Platelet count – G/L	11/134	257 [195–361 ]	251 [174–331]	0.080
Leucocyte count – G/L	11/29	9.60 [5.30–14]	9.50 [2.15–15.10]	0.757
Lymphocyte count – G/L	52/100	0.43 [0.09–0.89]	0.41 [0.11–0.86]	0.874

Results are presented as n(%) or median [interquartile range]

*ECMO* extracorporeal membrane oxygenation, *SOFA* Sequential Organ Failure Assessment, *PaO2* partial pressure of oxygen, *FiO2* fraction of inspired oxygen

### ECMO-associated infections

**Fig. 2:**
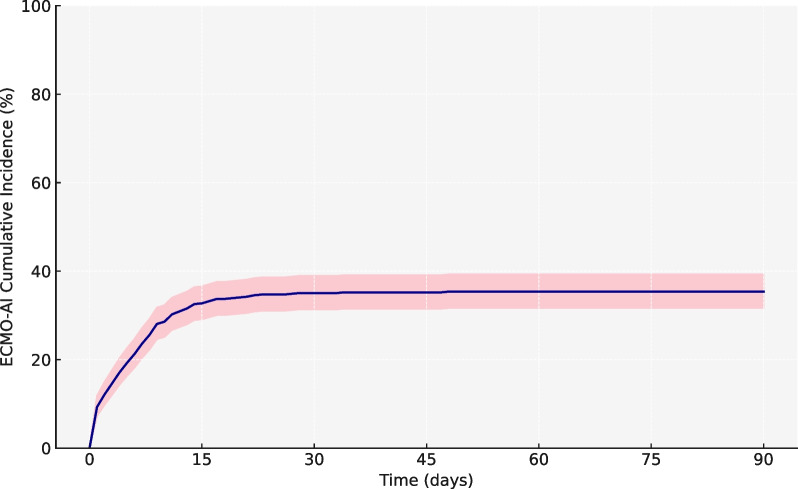
Cumulative ECMO-AI incidence

Overall, 214/602 patients (36%) experienced at least one ECMO-AI event. The incidence rate of ECMO-AI was 27 per 1000 ECMO days at risk (Fig. 2). VAP was diagnosed in 117 patients (incidence rate of 12 per 1000 ECMO days at risk) and BSI in 154 patients (incidence rate of 15 per 1000 ECMO days at risk). Additionally, 57/214 (27%) patients presented with both VAP and BSI. The time from cannulation to ECMO-AI was notably shorter for BSI compared to VAP, with medians of 4 (0–9) days and 5 (2–11) days, respectively (*p* = 0.017). The causative microorganisms are reported by infection site in Table 2 and Additional file 1: Figure S1. The main causative agents were Enterobacteriaceae (34% for BSI and 48% for VAP), Enterococcus species (25% and 6%, respectively) and non-fermenting Gram-negative bacilli (13% and 20%, respectively). Fungal infections were also noteworthy, with incidences of 10% for BSI and 3% for VAP. MDRO accounted for 21% and 15% of infections for BSI and VAP, respectively. The proportion of extended-spectrum beta-lactamase (ESBL) was 4% and 7%, respectively, and the proportion of methicillin-resistant Staphylococcus aureus (MRSA) was 3% and 3% respectively.

**Table 2:** Microorganisms responsible for ECMO associated infections by infection site

Microorganisms	Bloodstream infection (*n* = 154)	Ventilator associated Pneumonia (*n* = 117)	*P* value	Crude mortality (%)
Enterobacteriaceae	52 (33.8)	56 (47.9)	0.026	52
ESBL-PE	6 (3.9)	8 (6.8)	0.420	36
3rd generation cephalosporin-resistant	31 (20.1)	5 (12.8)	0.141	36
Enterococcus sp.	39 (25.3)	7 (6.0)	< 0.001	63
Vancomycin-resistant *Enterococcus species*	1 (0.6)	0 (0.0)	1.000	100
Non-fermenting Gram negative Bacilli	20 (13.0)	23 (19.7)	0.187	79
Imipenem-resistant *Acinetobacter sp.*	1 (0.6)	0 (0.0)	1.000	100
MDR *Pseudomonas sp.*	0 (0.0)	1 (0.9)	0.432	0
Coagulase negative Staphylococcus	35 (22.7)	4 (3.4)	< 0.001	54
Staphylococcus aureus	11 (7.1)	25 (21.4)	0.001	53
Methicillin-resistant *Staphylococcus aureus*	4 (2.6)	4 (3.4)	0.729	37
Fungi	15 (9.7)	4 (3.4)	0.075	58
Streptococcus sp.	5 (3.2)	6 (5.1)	0.641	36
Others	9 (5.8)	8 (6.8)	0.935	53
MDR microorganisms	33 (21.4)	18 (15.4)	0.270	41
Polymicrobial	31 (20.1)	16 (13.7)	0.219	55

Results are presented as n(%)

*ECMO* extracorporeal membrane oxygenation, *ESBL-PE* extended-spectrum beta-lactamase-producing Enterobacteriaceae, *MDR* multidrug resistant

*All VAP related to fungi were Pulmonary aspergillosis (*Aspergillus fumigatus* *n* = 3 *and Aspergillus sp.* *n* = 1) while all BSI related to fungi were Candidemia (*Candida albicans* *n* = 7 and *Candida sp.* *n* = 8)

### Outcomes

Crude mortality by microorganism and by infection site is reported in Table 2. The highest mortality rates were observed in patients with non-fermenting Gram-negative bacilli infection (79%) and with Enterococcus species infection (63%). Patients with ECMO-AI had longer ECMO support with a median of 16 (10–27) days, compared to 11 (5–19) days for those without ECMO-AI (*p* < 0.001) (Additional file 1: Table S2). Further analysis using a Cox multistate model (Additional file 1: Table S3) did not find an association between ECMO-AI and hospital death (HR = 1.00 95% CI [0.79–1.26], *p* = 0.986).

### Sensitivity analyses

We conducted a sensitivity analysis in which only ECMO-AI that developed after 48 h of ECMO run were considered. Patients with ECMO run < 48 h were excluded. We found that 157/546 patients (29%) acquired an ECMO-AI corresponding to an incidence rate of 21 ECMO-AI per 1000 ECMO-days. Outcomes were similar to those reported when considering the complete ECMO run (Additional file 1: Table S4). We also explored the potential for different patterns of early vs late ECMO-AI. We compared early (≤ 5 days from cannulation) and delayed (> 5 days from cannulation) ECMO-AI. Interestingly, there were no differences in microbiology nor in outcomes with respect for ECMO-AI timing (Additional file 1: Tables S5 and S6).

## Discussion

This study reported the incidence of ECMO-AI (defined as VAP and BSI during ECMO support) at a nationwide level in a large multicenter cohort of COVID-19 patients supported by ECMO. The main results were as follows. First, the incidence of ECMO-AI was high in this population, with 36% of patients and a rate of 27 ECMO-AI per 1000 ECMO days. Second, *Enterobacteriaceae* emerged as the main causative microorganisms. Third, we found a high incidence of *Enterococcus spp.* in BSI. Fourth, the incidence of MRSA and ESBL was low in our cohort. Finally, ECMO-AI were not associated with in-hospital death after multivariable analysis.

The incidence of ECMO-AI is highly variable across published observational studies, including the ELSO registry, ranging from to 9 to 65% [18]. Several factors contribute to this variability: the specific types of HAI considered in the analysis, the definitions employed and the underlying indications for ECMO. Diagnosing HAI on ECMO can be challenging, especially for cannulation site or catheter-associated urinary tract infections. Furthermore, distinguishing between colonization and infection may not always be definitive. Moreover, the mortality attributable to some infections, such as catheter-associated urinary tract infections, might be close to zero [19]. Consequently, the present study focused on the most common ECMO-AI, BSI and VAP, both of which have been shown to be associated with poorer outcomes in critically ill patients [20].

Regarding microbiology, we report here the most extensive description to date of the micro-organisms responsible for ECMO-AI. As observed in previous ECMO case series and in other critical-care settings, *Enterobacteriaceae* were the main causative microorganisms, found in a third of BSI and almost half of VAP [1, 14, 21, 22]. *Enterobacteriaceae* also predominated in VAP and BSI in critically COVID-19 patients [2, 3, 5]. Similarly, non-fermenting Gram-negative bacilli were highly represented in VAP (20%) in our cohort, in line with previous publications involving both COVID-19 and non-COVID-19 critically ill patients [1–5].

Strikingly, a high proportion of *Enterococcus spp.* were reported in BSI cases (25%), which was unexpected. Recently, the international EUROBACT-2 study, encompassing 2,927 hospital-acquired BSI episodes in non-COVID-19 patients, reported 314 *Enterococcus spp.* infections (11%), much lower than observed in the present study. Regarding non-COVID-19 ECMO patients, previous case series also reported lower proportions of *Enterococcus spp*. BSI, ranging from 15 to 20% [1, 22]. Several factors may explain this difference. First, *Enterococcus spp*. was frequently identified in BSI cases in COVID-19 patients, such as reported in Spain (30%), in Italy (25%) or in France (15%) (2, 4, 23). In our cohort, the majority of critically ill COVID-19 patients received antimicrobial agents at admission, primarily cephalosporins, which may have promoted *Enterococcus spp*. proliferation and subsequent translocation [23, 24]. Furthermore, cross-transmission of *Enterococcus spp*. has been frequently observed, especially in high-activity ICUs as observed during the pandemic [25]. Notably, this microorganism was only identified in a few cases (6%) of VAP. The implications of Enterococcus respiratory colonization, or even infection, remain controversial, and identification in respiratory sample is usually dismissed as contamination. Finally, MDRO were identified in nearly 20% of ECMO-AI in our cohort, with low levels of MRSA or ESBL. The EUROBACT-2 study reported a similar 22% rate of difficult-to-treat Gram-negative bacteria. However, in this study, the prevalence of resistant Gram-positive bacteria was higher at 37%, compared to 3% in our study [14]. For critically ill COVID-19 patients, another large French cohort reported higher prevalence of MDRO with up to 30% resistance to 3rd Generation Cephalosporin and 17% of ESBL in Enterobacteriaceae and 11% of MRSA [4]. Similarly, an European cohort of COVID-19 critically ill patients found high rates of MDR [26].

Interestingly, we found a high incidence of fungal infection in our population, a proportion much higher than previously described in non-COVID-19 ECMO patients [27].

ECMO-AI were not found associated with mortality in our cohort, in line with previous study which reported that HAI do not modify outcome in the most severe patients such as those with ECMO support [28]. Interestingly, ECMO-AI were associated with length of ECMO support, length of mechanical ventilation and length of ICU stay in bivariate analysis. This is likely related in part to the duration of exposure, i.e., longer ECMO exposure creates more opportunities for ECMO-AI. The other potential effect is that ECMO-AI may delay decannulation or extubation and prolong ICU stays.

Our study has several strengths. First, our cohort is one of the largest samples of COVID-19 patients supported by ECMO, providing detailed microbiological data on ECMO-HAI. Second, the participating centers cover a majority of the available ECMO sites in France. Third, the multicenter design facilitates the generalizability of our findings. Finally, the database's quality was regularly assessed by dedicated data managers.

However, there are limitations to consider. Despite wide representation, not all French ECMO centers were included, potentially introducing selection bias. Further, being an observational study, this study might be subject to information bias. The absence of specific HAI prevention recommendations might result in variations in the prevention practice across the ICUs. Additionally, as mentioned above, we focused on VAP and BSI and we do not provide information on catheter-related urinary tract infections or cannulation site infections. Moreover, the source of BSI was not recorded in our database. As both ECMO cannulation itself and patient illness severity at cannulation contribute to the development of ECMO-AI, we classified as ECMO-AI any infection occurring during the entire ECMO run. However, alternative definitions exist in the literature, which consider different exposure periods for ECMO-AI [29]. Finally, most of our patients (75%) were included during the first wave of the pandemic in a context of work overload and bed shortage which may have resulted in difficulties to maintain adequate preventive measures.

## Conclusions

In conclusion, our study demonstrated a high incidence of ECMO-AI in a nationwide multicenter cohort of patients with severe COVID-19 supported with ECMO. *Enterobacteriaceae* were the main causative microorganisms, with low rates of ESBL and MRSA. ECMO-AI were not found associated with in-hospital mortality.

### Supplementary Information

**Additional file 1 **of Healthcare-associated infections in patients with severe COVID-19 supported with extracorporeal membrane oxygenation: a nationwide cohort study.

## Data Availability

The dataset analyzed during the current study is available from the corresponding author on reasonable request.

## Abbreviations

BSI: Bloodstream Infections
BMI: Body mass index
COVID-19: Coronavirus disease 2019
ESBL: Extended-spectrum beta-lactamase
ECMO: Extracorporeal membrane oxygenation
ECMO-AI: ECMO-associated infections
HAI: Healthcare associated infections
IRR: Incidence rate ratio
MDRO: Multidrug-resistant organisms
MRSA: Methicillin-resistant Staphylococcus aureus
SARS-CoV-2: Severe acute respiratory syndrome coronavirus 2
SOFA: Sequential organ failure assessment
VAP: Ventilator-associated pneumonia
VA-ECMO: Veno-arterial ECMO
VV-ECMO: Veno-venous ECMO
