# Supplementary material for: Healthcare-associated infections in patients with severe COVID-19 supported with extracorporeal membrane oxygenation: a nationwide cohort study
Source: Crit Care. 2024 Feb 20;28:54. doi: 10.1186/s13054-024-04832-3 (PMC10877839; doi:10.1186/s13054-024-04832-3)

**Table S1- Definition of variables**

| Variables | Definitions |
| --- | --- |
|  |  |
| Chronic respiratory failure | Arterial pO2 on room air less than 60 mmHg for 3 months or more |
| Chronic kidney disease | Glomerular filtration rate <60 mL/min/ 1.73 m2 for 3 months or more |
| Onco-hematological malignancy | Ongoing carcinologic treatment |
| ARDS | Berlin Criteria for ARDS. JAMA. 2012;307(23):2526-2533. doi:10.1001/jama.2012.5669 |
| SAPS II | Simplified Acute Physiology Score II according to Le Gall et al. PMID: 8254858 DOI: 10.1001/jama.270.24.2957 |
| SOFA score | Sequential-related Organ Failure Assessment score, according to Vincent et al. PMID: 8844239 DOI: 10.1007/BF01709751 |
| Thrombotic complications on ECMO | One or more of the following complications: ischemic stroke, deep vein thrombosis, pulmonary embolism or thrombosis, acute mesenteric ischemia, acute coronary syndrome, acute limb ischemia, macroscopic thrombus of circuit/membrane without needing to change the circuit, oxygenator failure requiring change due to clot formation, acute circuit thrombosis requiring change |
| Acute kidney injury on ECMO | Acute kidney injury according to KDIGO classification kidney International Supplements (2012) 2, 8–12; doi:10.1038/kisup.2012.7 |

**Table S2- Outcomes of patients with or without ECMO-associated infections**

|  | No ECMO-associated infection  (n=388) | ECMO-associated infection  (n=214) | P-value |
| --- | --- | --- | --- |
| Acute kidney injury on ECMO (missing data 6/0) | 180 (47) | 104 (48.6) | 0.794 |
| Renal replacement therapy | 49 (13) | 26 (12) | 0.161 |
| Thrombotic complications on ECMO (missing data 3/1) | 275 (71) | 116 (55) | <0.001 |
| Limb ischemia (missing data 6/0) | 10 (3) | 2 (1) | 0.228 |
| Length of mechanical ventilation duration – days | 23 [12-38] | 28 [19-43] | <0.001 |
| Length of ECMO support – days | 11 [5-19] | 16 [10-27] | <0.001 |
| Length of ICU stay – days | 27 [13-43] | 33 [22-48] | <0.001 |
| ECMO-free days within 90 days of cannulation – days | 0 [0-58] | 0 [0-64] | 0.109 |
| Ventilatory-free days within 90 days of cannulation – days | 0 [0-39] | 0 [0-49] | 0.023 |
| In hospital death | 226 (58) | 115 (54) | 0.326 |

Results are presented as n(%) or median [interquartile range]. ECMO: extracorporeal membrane oxygenation, ICU: intensive care unit *Missing data n=6

**Table S3 - Risk factors for in-hospital death**

| Variables | HR ^a^ | 95% CI ^a^ | P-value ^a^ |
| --- | --- | --- | --- |
| Epidemic waves |  |  |  |
| First epidemic wave | – | – | – |
| Subsequent epidemic waves | 1.37 | 1.05-1.79 | 0.022 |
| Age (per one year) | 1 | 1-1.01 | 0.058 |
| Center case-volume |  |  |  |
| High | c | – | – |
| Intermediate | 1.27 | 0.94-1.72 | 0.122 |
| Low | 0.97 | 0.58-1.6 | 0.891 |
| Comorbidities |  |  |  |
| Diabetes | 1.27 | 1-1.6 | 0.049 |
| Chronic respiratory failure | 1.34 | 0.74-2.41 | 0.333 |
| Chronic kidney failure | 0.91 | 0.53-1.56 | 0.729 |
| Onco-hematological malignancy | 1.64 | 0.84-3.19 | 0.144 |
| Septic shock | 1.31 | 0.96-1.78 | 0.09 |
| Treatment before cannulation |  |  |  |
| Steroids | 0.8 | 0.52-1.25 | 0.325 |
| NSAIDs | 0.59 | 0.31-1.1 | 0.096 |
| Antibiotics | 0.95 | 0.64-1.42 | 0.808 |
| Selective digestive decontamination | 1.58 | 0.87-2.89 | 0.135 |
| Delay from intubation to cannulation (per one day) | 1 | 0.98-1.02 | 0.802 |
| Type of ECMO |  |  |  |
| Veno-venous | – | – | – |
| Veno-arterial | 1.69 | 1.13-2.50 | 0.009 |
| SOFA score (per 1 point increment) | 1.07 | 1.04-1.11 | <0.001 |
| **ECMO associated infection** | 1.00 | 0.79-1.26 | 0.986 |

Results are presented as hazard ratio (HR) with 95% confidence interval (95% CI). ECMO: extracorporeal membrane oxygenation; SOFA: Sequential Organ Failure Assessment; PaO2: partial pressure of oxygen; FiO2: fraction of inspired oxygen; NSAIDs: Nonsteroidal anti-inflammatory drugs; SOFA: Sequential Organ Failure Assessment. ^a^ Multistate multivariable Cox proportional-hazards model adjusted for predefined confounders based on ECMO and COVID-19 literature. Multivariable model was applied after multiple imputation.

**Table S4- Outcomes of patients with or without ECMO-associated infections that developed after 48 h of ECMO run**

|  | No ECMO-associated infection  (n=389) | ECMO-associated infection  (n=157) | P-value |
| --- | --- | --- | --- |
| Acute kidney injury on ECMO (missing data 4/0) | 179 (47) | 72 (46) | 0.969 |
| Renal replacement therapy | 163 (42) | 56 (36) | 1.000 |
| Thrombotic complications on ECMO (missing data 2/1) | 264 (68) | 82 (52) | 0.001 |
| Limb ischemia (missing data 4/0) | 7 (2) | 2 (1) | 1.000 |
| Length of mechanical ventilation duration – days | 25 [15-41] | 29 [21-43] | 0.011 |
| Length of ECMO support – days | 12 [8-22] | 16 [10-27] | 0.002 |
| Length of ICU stay – days | 30 [17-47] | 36 [24-49] | 0.005 |
| ECMO-free days within 90 days of cannulation – days | 0 [0-62] | 16 [0-64] | 0.122 |
| Ventilatory-free days within 90 days of cannulation – days | 0 [0-43] | 11 [0-51] | 0.024 |
| In hospital death | 211 (54) | 80 (51) | 0.547 |

Results are presented as n(%) or median [interquartile range]. ECMO: extracorporeal membrane oxygenation, ICU: intensive care unit

**Table S5- Outcomes of patients with early vs late ECMO-associated infections**

|  | Early ECMO-associated infection (≤ 5days from cannulation)  (n=116) | Late ECMO-associated infection (> 5 days from cannulation)  (n=98) | P-value |
| --- | --- | --- | --- |
| Acute kidney injury on ECMO | 64 (55) | 40 (41) | 0.050 |
| Renal replacement therapy | 15 (13) | 8 (8) | 0.368 |
| Thrombotic complications on ECMO (missing data 1/0) | 68 (59) | 48 (49) | 0.179 |
| Limb ischemia | 2 (2) | 0 (0) | 0.501 |
| Length of mechanical ventilation duration – days | 26 [17-39] | 32 [24-46] | 0.040 |
| Length of ECMO support – days | 13 [8-24] | 18 [12-29] | 0.050 |
| Length of ICU stay – days | 31 [19-46] | 36 [25-50] | 0.083 |
| ECMO-free days within 90 days of cannulation – days | 0 [0-70] | 13 [0-60] | 0.779 |
| Ventilatory-free days within 90 days of cannulation – days | 0 [0-51.25] | 7 [0-46] | 0.644 |
| In hospital death | 64 (55) | 51 (52) | 0.749 |

Results are presented as n(%) or median [interquartile range]. ECMO: extracorporeal membrane oxygenation, ICU: intensive care unit

**Table S6 - Microorganisms responsible for early *vs* late ECMO-associated infections**

| Microorganisms | Early ECMO-associated infection  (≤ 5 days from cannulation)  (n=141) | Late ECMO-associated infection  (> 5days from cannulation)  (n=130) | P-value |
| --- | --- | --- | --- |
| Enterobacteriaceae | 61 (43.3) | 47 (36.2) | 0.285 |
| ESBL-PE | 6 (4.3) | 8 (6.2) | 0.667 |
| 3rd generation cephalosporin-resistant | 22 (15.6) | 24 (18.5) | 0.627 |
| Enterococcus sp. | 18 (12.8) | 28 (21.5) | 0.078 |
| Vancomycin-resistant *Enterococcus species* | 0 (0.0) | 1 (0.8) | 0.480 |
| Non-fermenting Gram negative Bacilli | 24 (17.0) | 19 (14.6) | 0.708 |
| Imipenem-resistant *Acinetobacter sp.* | 1 (0.7) | 0 (0.0) | 1.000 |
| MDR *Pseudomonas sp.* | 1 (0.7) | 0 (0.0) | 1.000 |
| Coagulase negative Staphylococcus | 26 (18.4) | 13 (10.0) | 0.071 |
| Staphylococcus aureus | 19 (13.5) | 17 (13.1) | 1.000 |
| Meticillin-resistant *Staphylococcus aureus* | 4 (2.8) | 4 (3.1) | 1.000 |
| Fungi | 9 (6.4) | 10 (7.7) | 0.854 |
| Streptococcus sp. | 6 (4.3) | 5 (3.8) | 1.000 |
| Others | 9 (6.4) | 8 (6.2) | 1.000 |
| MDR microorganisms | 25 (17.7) | 26 (20.0) | 0.747 |
| Polymicrobial | 31 (22.0) | 16 (12.3) | 0.052 |

Results are presented as n(%). ECMO: extracorporeal membrane oxygenation; ESBL-PE: extended-spectrum beta-lactamase-producing Enterobacteriaceae; MDR: multidrug resistant.

**Figure S1: Microorganisms by infection site**

VAP: ventilator-associated pneumonia; BSI: bloodstream infection.


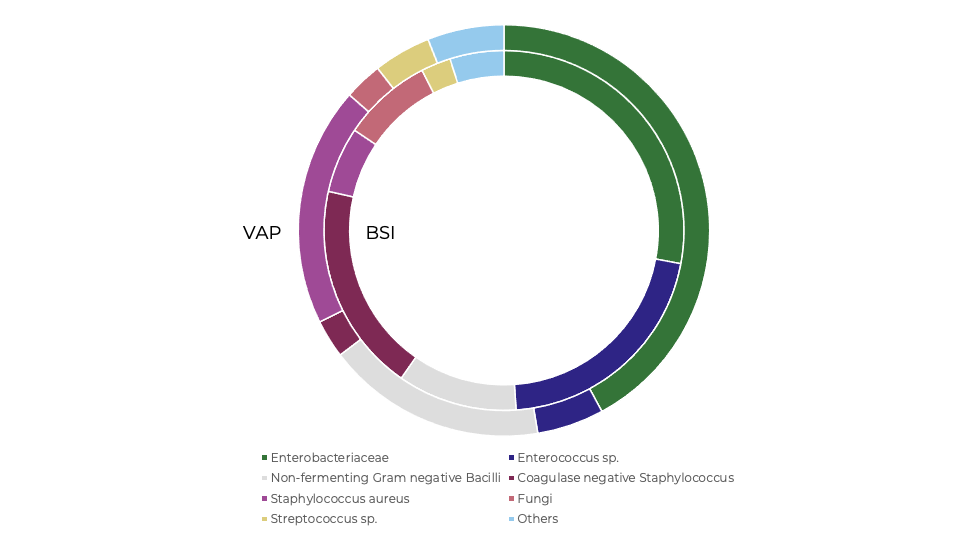

Supplement: Supplementary file 1 — Additional file 1 of Healthcare-associated infections in patients with severe COVID-19 supported with extracorporeal membrane oxygenation: a nationwide cohort study. [file 13054_2024_4832_MOESM1_ESM.docx]
